# How to optimize switch virtual keyboards to trade off speed and accuracy

**DOI:** 10.1186/s41235-016-0007-6

**Published:** 2016-09-22

**Authors:** Xiao Zhang, Kan Fang, Gregory Francis

**Affiliations:** 1 Department of Computer Science, Purdue University, West Lafayette, 47907-2004 IN USA; 2 College of Management and Economics, Tianjin University, Tianjin, 300072 China; 3 Department of Psychological Sciences, Purdue University, West Lafayette, 47907-2004 IN USA

**Keywords:** Switch virtual keyboard, Mixed integer programming, Locked-in patients, Motor disability

## Abstract

In some circumstances, people interact with a virtual keyboard by triggering a binary switch to guide a moving cursor to target characters or items. Such switch keyboards are commonly used by patients with severely restricted motor capabilities. Typing with such systems enables patients to interact with colleagues, but it is slow and error prone. We develop a methodology that can automate an important part of the design process for optimally structured switch keyboards. We show how to optimize the design of simple switch keyboard systems in a way that minimizes the average entry time while satisfying an acceptable error rate. The first step is to model the user’s ability to use a switch keyboard correctly for different cursor durations. Once the model is defined, our optimization approach assigns characters to locations on the keyboard, identifies an optimal cursor duration, and considers a variety of cursor paths. For our particular case, we show how to build a user model from empirical data and demonstrate that the resulting optimized keyboards are quite different from existing keyboard designs.

**Electronic supplementary material:**

The online version of this article (doi:10.1186/s41235-016-0007-6) contains supplementary material, which is available to authorized users.

## Significance

Patients with severe motor disability often use a switch keyboard, where binary actions from the user guide a moving cursor to items on the keyboard. Such switch devices enrich patients’ lives by allowing them to communicate with friends, family, and work colleagues. However, such devices are notoriously slow and even small benefits in performance can dramatically improve communication. We show how to measure and model human performance with a switch device in a way that enables the application of optimization algorithms to design switch keyboards that minimize entry time while satisfying a defined acceptable error rate. The resulting optimal designs are quite different from existing products.

## Background

Computers play an integral role in the social and economic lives of most people, but some people are unable to use a traditional keyboard or touchscreen. For example, some spinal or brain injuries lead to locked-in syndrome where patients are almost fully paralyzed and cannot speak, even though their cognitive abilities are intact (Laureys et al., 2005). Such patients have very limited interaction with their environment, and computer access can greatly enhance their quality of life. To provide computer access, human–computer interactions leverage the limited muscle control (e.g., an eye blink or a small muscle contraction) of the user to trigger binary signals in switch devices. As described in detail below, such devices work with a virtual keyboard to guide a cursor on the keyboard to select a desired character or function. This type of switch keyboard thereby allows patients to engage in local and on-line discussions with caregivers, friends, and employers (Bauby, 1997; Tavalaro & Tayson, 1997).

Several virtual keyboard designs have been proposed by commercial companies. For example, the SwitchXS keyboard provides various modes that implement mouse input and keyboard characters (AssistiveWare, 2013). In a common design, an initial switch activation initiates a cursor that follows a path across the keyboard and stops to select an item when the user triggers the switch device again. A default approach is for the user first to select a row containing a desired item and thereby direct the cursor to move across that row’s items for an additional user selection. Other keyboard designs use a similar strategy but define differently the groups of items that can be selected. For example, in the Logo keyboard (Norte & Lobo, 2007), two major scanning groups (numeric and alphabetic) are displayed, and the user first triggers the switch to select one of these two groups, and then selects the item in the selected group as the cursor moves across the group.

Because of the very limited type of user input (only a binary switch), the cursor must scan (or be guided by switch actions) across different keyboard items. Indeed, these types of systems are sometimes referred to as scanning keyboards (MacKenzie & Felzer, 2010). Such an approach means that some items are reached relatively quickly (those near the beginning of the cursor path) while other items take longer to select (those near the end of the cursor path). Thus, the arrangement of characters along the path significantly affects input efficiency, and many studies have explored ways to improve the design of virtual keyboards (not only for patients, but also for other specialized keyboards). A simple and effective way to improve keyboard efficiency is to place commonly used keyboard items early in the cursor path (Hughes, Warren, & Buyukkokten, 2002; Mayzner & Tresselt, 1965; Zhai, Hunter, & Smith, 2002).

Other factors also influence the usability of a virtual keyboard. For example, the scanning speed of the cursor cannot be too fast (users would not have enough time to respond correctly) nor too slow (users would have to wait unnecessarily for the cursor to reach a desired item). Francis and Johnson (2011) showed that character placement on a switch virtual keyboard can be treated as a mathematical optimization problem with a trade-off between speed and accuracy. They also proposed an algorithm to design a keyboard that optimized the cursor speed for a given desired accuracy.

**Fig. 1:**
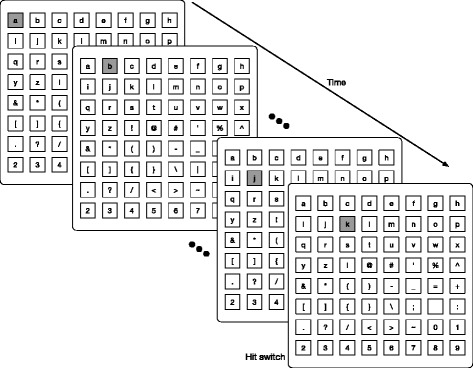
Linear cursor path keyboard. In a linear cursor path, the cursor moves key by key across the keyboard. In this example, the cursor starts at the *upper left corner* and moves in a wrap-around pattern across the keyboard rows. Thus, it would move from the “h” key at the right side of row 1 to the “i” key at the left side of row 2. When the cursor is over the desired key, the user triggers the switch to select that key (here, the letter “k”). To simplify the display, movement of the cursor is not explicitly shown between the “b” key and the “j” key

An important component of keyboard optimization is the path the cursor follows across the keyboard. There are many different possible cursor scan paths (MacKenzie & Felzer, 2010; MacKenzie 2012). Figure 1 shows a keyboard where the cursor follows a linear cursor path (see Additional file 1: Movie 1 for an animated version). Here, the cursor starts at the first key (top left) and then proceeds to each key one by one by wrapping from the end (right side) of one row to the beginning (left side) of the next row. When the cursor covers the key of interest, the user triggers the switch and thereby selects that item. With this kind of cursor path, selecting any virtual key requires only one action by the user, and 1 to 64 cursor steps.

**Fig. 2:**
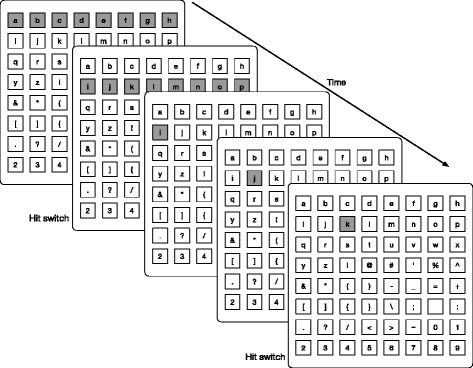
Row-column cursor path keyboard. In a row–column cursor path, the cursor moves across rows until the switch is triggered and then moves across the keys in the selected row (here, row 2). A trigger of the switch selects the desired key (here, the third key, to select “k”)

Figure 2 (Additional file 2: Movie 2) demonstrates a row–column cursor path, which is commonly used in switch keyboard designs. Here, the cursor starts at the top row and first scans across the rows to allow the user to select the row containing the target character. When a row is selected, the cursor then scans the columns in the selected row so that the user is able to select the target key by triggering the switch device again. With this kind of cursor path, selecting any virtual key requires two actions by the user and two to 16 cursor steps.

**Fig. 3:**
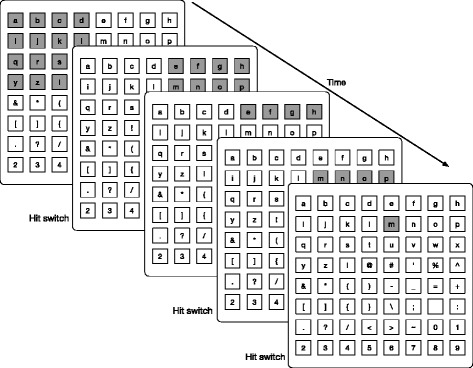
Quadrant cursor path keyboard. In a quadrant cursor path, the cursor moves across different quadrants of the keyboard, then across rows in the selected quadrant, and then across the keys in the selected row. In the example shown, it takes five cursor steps and three switch actions to select the “m” key

Figure 3 (Additional file 3: Movie 3) shows the same kind of keyboard where the cursor follows a quadrant cursor path. Here the cursor first moves across the four quadrants of the keyboard. When the user triggers the switch, the cursor follows a row–column path within the selected quadrant. With this kind of cursor path, selecting any virtual key requires three selections by the user (one for the quadrant, one for the row, and one for the column), and three to 12 cursor steps.

**Fig. 4:**
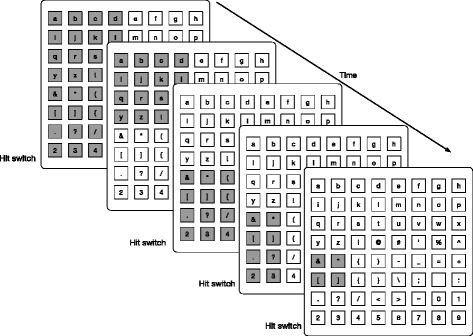
In a binary cursor path, the cursor segments the keyboard into remaining halves. The user triggers the switch to select the keyboard half that contains the target. The selected region is then halved again for an additional selection. The process continues until the cursor is on the target key. In the example shown, the user is guiding the cursor toward the “*” key, and three more cursor steps and two more switch selections from the user are required

Figure 4 (Additional file 4: Movie 4) shows the same kind of keyboard where the cursor follows a binary cursor path. Here, the cursor first moves across the left and right halves of the keyboard. A selection by the user focuses the cursor path to the selected side, which is then divided into a top and bottom. Further selections keep dividing the number of remaining keys in half and guide the cursor toward the target key. With this kind of cursor path, selecting any virtual key requires six selections by the user and six to 12 cursor steps.

As Figs. 1, 2, 3 and 4 and Additional file 1: Movie 1, Additional file 2: Movie 2, Additional file 3: Movie 3, and Additional file 4: Movie 4 demonstrate, different cursor paths can be assigned to the very same physical layout of the keyboard. In terms of guiding the cursor to a target key, it is the cursor path, rather than the physical layout of keys, that determines the efficiency of the keyboard. To enable users to learn and remember the cursor path, the physical layout of the keyboard may reflect the groupings of virtual keys along the path, but our analysis supposes that users know the cursor path regardless of its complexity.

The choice of a cursor path is important because it imposes requirements on the user and determines the time needed to select an item. For example, accessing a key on the keyboard for the linear cursor path requires only one selection from the user, while the binary cursor path requires six selections. If triggering the switch device is difficult for the user, it might be best to use a cursor path that needs few selections. On the other hand, the linear cursor path will take a long time to reach items at the end of the path (64 cursor steps for the final item), while the binary cursor path takes no more than 12 cursor steps. Several studies have explored the costs and benefits of different types of cursor paths (e.g., Koester & Simpson, 2014).

The need for severely disabled patients to communicate is so striking that researchers are continually exploring methods to improve their performance (e.g., Broderick & MacKay, 2009; Koester & Simpson, 2014; Lin, Wu, Chen, Yeh, & Wang, 2008; MacKenzie & Felzer, 2010; Miró-Borrás & Bernabeau-Solar, 2009; Morland, 1983; Sears & Zha, 2003). A challenge for these investigations is that different approaches require development of new tools for applying design constraints related to a particular instance. Here, we provide a general approach that can be modified to consider other constraints and aspects of switch keyboard designs.

Because it formed the motivation for the basic optimization approach, in this paper we focus on the optimization of a switch virtual keyboard design by considering a variety of factors that influence the speed and accuracy of a keyboard. The rest of the paper is organized as follows. The next section quantitatively describes the keyboard design problem and develops a mixed integer programming (MIP) algorithm that solves a key part of the design problem. This algorithm is orders of magnitude faster than one described in Francis and Johnson (2011), and many of the subsequent results are tractable only because the MIP algorithm provides a quick solution to a critical part of keyboard design optimization. The subsequent section describes a behavioral experiment that measures performance for a switch keyboard. The subsequent section explains how to develop a performance model from the behavioral data. With the resulting model, the final section describes optimized keyboards for several different situations and compares and contrasts different keyboard designs. The experimental data files, analysis scripts, and optimization programs are available at the Open Science Framework (https://osf.io/vuaxj/?view_only=489c5b2f9b8b45cbb866416ef1e50ef4).

### An algorithm to solve the speed/accuracy trade-off

We describe the design problem in a general way because it demonstrates the common aspects of keyboard design for many different situations. An instance of the virtual keyboard design problem consists of an integer set $\mathcal {I}=\{1,2,\dots,N\}$ that refers to characters and an integer set $\mathcal {K}=\{1,2,\dots,N\}$ that refers to keyboard locations. The design task is to assign the characters to keyboard locations. Let *F*_*i*_ be the frequency of the *i*-th character in a given text corpus. These character frequencies might be estimated from general databases, or might be based on the specific type of text entered by a specific user.

First consider the time needed to reach a location on the keyboard (speed of entry). Let *S*_*k*_ be the number of cursor steps required to reach location *k* from the start of the cursor path. For example, for the row–column cursor path shown in Fig. 2, to reach position 12 (supposing the keys are indexed in sequence from left-to-right and top-to-bottom), it will first take two steps to reach the second row, and then four steps to reach the fourth column in row 2. Therefore, in this cursor path, the total number of steps to reach position 12 is *S*_12_=2+4=6. Note that the value of *S*_*k*_ depends on the cursor path; different cursor paths may lead to quite different values of *S*_*k*_ even for the same key position. In addition, we let *D* be the duration that a cursor will stay on each step of the path. Therefore, the time to reach position *k* is *D*×*S*_*k*_.

Now consider the accuracy of reaching a location on a keyboard. Let *P*_*k*_ be the probability of the user making an error while trying to guide the cursor to position *k*. It is reasonable to imagine that *P*_*k*_ increases as *D* decreases (with a slower cursor the user is less likely to make an error), but the exact nature of the relationship needs to be measured or modeled. We will measure and model the relationship in later sections.

To promote subsequent calculations, we introduce an indicator variable *X*_*ik*_ that is equal to 1 if the *i*-th character is assigned to position *k* and 0 otherwise. Then we define the *average entry time* across all entries as

1 $$ C_{t}= \frac{\sum\limits_{i\in \mathcal{I}}\sum\limits_{k\in \mathcal{K}}F_{i}DS_{k} X_{ik}}{\sum\limits_{i\in \mathcal{I}}F_{i}}  $$

where most of the terms in the numerator summation will be zero, except for where item *i* is at position *k*. At those positions, the numerator sums the entry time (*D* *S*_*k*_) multiplied by the frequency, *F*_*i*_, of character *i*. We also define the *average error rate* as

2 $$ C_{e}=\frac{\sum\limits_{i\in \mathcal{I}}\sum\limits_{k \in \mathcal{K}}F_{i}P_{k}X_{ik}}{\sum\limits_{i\in \mathcal{I}}F_{i}},  $$

which gives the average error probability across all character entries.

In general, *C*_*t*_ and *C*_*e*_ trade off each other. For example, placing the most commonly used characters near the beginning of the cursor path will decrease *C*_*t*_, but if those locations are error prone, then *C*_*e*_ will increase. We suggest that a practical way to trade off speed and accuracy is to identify a user-defined acceptable error rate, *ε*. We then describe the problem as assigning characters to the keyboard so that *C*_*e*_≤*ε* and *C*_*t*_ is minimized.

Another practical constraint on keyboard design is that some groups of characters should be spatially grouped because of historical reasons or user preferences. For example, the numerical characters (i.e., 0–9) are commonly co-located and ordered. For the keyboards considered here, we grouped them together and put them in the tail of the keyboard layout, that is, the numerical characters are assigned to locations as follows:

3 $$\begin{array}{@{}rcl@{}} \mathcal{G} &=\{(55, 55), (56, 56), (57, 57), (58, 58), (59, 59),\\ &\quad (60, 60), (61, 61), (62, 62), (63, 63), (64, 64)\}\notag \end{array} $$

where the indices 55–64 for the first coordinate correspond to the numerical characters 0–9 and the indices 55–64 for the second coordinate indicate the last ten locations on the keyboard. Figures 1, 2, 3, and 4 (and Additional file 1: Movie 1, Additional file 2: Movie 2, Additional file 3: Movie 3, and Additional file 4: Movie 4) show keyboards that follow these constraints.

We now describe how to find (Pareto) optimal solutions in which no other solution has both a lower average entry time and satisfactory error rate. The task can be modeled as a MIP problem:

4a $$\begin{array}{*{20}l} \text{minimize}~ &C_{t}&&  \end{array} $$

4b $$\begin{array}{*{20}l} \text{subject to}~ & \sum\limits_{k\in \mathcal{K}}X_{ik}=1, &&\text{for all}\ i\in\mathcal{I}; \end{array} $$

4c $$\begin{array}{*{20}l} &\sum\limits_{i\in \mathcal{I}}X_{ik}=1, &&\text{for all}\ k\in\mathcal{K}; \end{array} $$

4d $$\begin{array}{*{20}l} &X_{ik}=1, &&\text{for each}~ (i, k)\in\mathcal{G};  \end{array} $$

4e $$\begin{array}{*{20}l} &C_{e} \le \varepsilon,  \end{array} $$

4f $$\begin{array}{*{20}l} &X_{ik} \in \{0, 1\}, &&\text{for each}\ i\in\mathcal{I}; k\in\mathcal{K}; \end{array} $$

4g $$\begin{array}{*{20}l} &C_{t}, C_{e} \ge 0. && \end{array} $$

Constraint (4a) seeks to minimize the average time needed to reach items on the keyboard. Constraint (4b) ensures that each character can be assigned to only one position on the keyboard. Constraint (4c) ensures that each position in the keyboard layout contains only one character. Constraint (4d) determines the arrangement of the numerical characters as mentioned above. Constraint (4e) ensures that the average error rate is no larger than the user-identified acceptable error rate *ε*. Constraints (4f) and (4g) are variable-type constraints that ensure the objective functions remain true to their definitions.

As an initial check on the MIP approach, we took the *S*_*k*_, *F*_*i*_, *D*, and *P*_*k*_ terms as defined by Francis and Johnson (2011) and used Gurobi Optimizer 5.6 (Gurobi, 2013) to solve the MIP problem. The resulting solution of characters assigned to locations on the keyboard was very similar to what was produced by Francis and Johnson (2011) with a hill-climbing algorithm. However, the MIP algorithm was much faster. It took approximately 3 seconds to find a solution while the hill-climbing algorithm took approximately 3 h to generate essentially the same keyboard layout. Furthermore, the MIP algorithm is guaranteed to find an optimal keyboard layout that satisfies the acceptable error rate condition (if one exists), while the hill-climbing algorithm yields a keyboard layout that may not be optimal.

For any given keyboard design task, most of the terms in the MIP model are readily available: the cursor duration, *D*, can be taken as a given value for any instance; the character frequencies, *F*_*i*_, can be estimated from a text corpus; the number of cursor steps, *S*_*k*_, can be calculated for a given cursor path; and characters assigned to fixed positions, $\mathcal {G}$, can be readily created as needed. The only terms that remain to be identified are the error probabilities for different keyboard positions, *P*_*k*_. In the next section, we describe how to estimate these terms with a behavioral study.

### Estimates of error probabilities

This section demonstrates one way to estimate the *P*_*k*_ terms that are needed to drive the optimization approach described above. *P*_*k*_ corresponds to the probability that a user guiding the cursor to key *k* on a switch keyboard makes an error some place along the cursor path to that keyboard location. Ideally, these values would be guided by basic research on how quickly and reliably humans respond to dynamic stimuli. Although the literature on reaction time, timing, and predicted movements is enormous (Jensen, 2006; Posner, 1978), we were unable to identify published work that provides a model framework for characterizing the probability of correct responses for the kind of task involved in using a switch keyboard. Speed/accuracy trade-offs are commonly studied in areas such as traditional typing (Yamaguchi, Crump & Logan, 2013), but there the users largely control their actions rather than time their action to coincide with a stimulus event (the cursor being over an appropriate section of the keyboard). Likewise, the switch keyboard task seems related to simple reaction times, but effective use of the keyboard involves planning a precisely timed action rather than quickly responding to a stimulus. Such plans may occur well before the cursor covers a relevant part of the keyboard. Perhaps the area of basic research closest to the actions involved in a switch keyboard are measures of coincidence timing (Smith & McPhee, 1987). Even here, though, the fit is not perfect, as the cursor movements are highly learned and initiated by switch keyboard users. Ultimately, we suspect that switch keyboard users memorize a planned set of timed actions that are learned for commonly used characters with a specific cursor duration (for example, see the user video at http://www.assistiveware.com/a-pivotal-role-in-the-household).

Here we describe an experiment that estimates these error probabilities for the different locations of the keyboard for several different cursor durations. These estimates will both contribute directly to the optimization methods described above and provide basic research into the accuracy of a sequence of timed actions. For the design task, the ideal experiment would estimate these probabilities from the same kind of individuals who will ultimately use the switch keyboard. In many cases, the ultimate users are patients with severe motor disabilities and their performance with a switch device might be quite different from a non-patient population. To complicate matters further, performance on the switch keyboard should be estimated after users have had substantial experience using the switch keyboard; otherwise the estimated probabilities will become inaccurate as users improve with additional practice.

With these constraints in mind, it seemed impractical (and perhaps unjustifiable) to ask patients to participate in a tedious experiment to measure performance on a switch keyboard until we had demonstrated the validity and value of the overall optimization approach. Thus, rather than using locked-in patients, we recruited seven students from Purdue University to complete up to 25 experimental sessions. The use of normal subjects rather than patients with motor disabilities restricts our conclusions about which keyboards should be used for a given person, but this was always going to be a conclusion of this kind of study. The optimized keyboard design for a given patient will depend on the characteristics of the user, their familiarity with the device, the type of text they enter, and their acceptable error rate. Since our intention is to demonstrate an optimization algorithm that can accommodate these individual characteristics, gathering data from normal students is appropriate (and much easier). Of course, interesting optimization outcomes may also be derived by specifically studying the characteristics of patients.

### Subjects

Seven student participants used a button-style switch device (Origin Instruments, 2013) that is similar to a computer mouse, but specially designed for people with motor disabilities that use switch keyboards. A participant could attend at most two sessions each day (in the morning and afternoon, respectively). Before the formal experiments, a 1 h tutorial was given so that the participant could practice and get familiar with the keyboard and the switch devices. To motivate participants to perform their best, each participant received a base of $5 for each session and a reward of $0.025 for each correct character entry. On average, participants earned around $45 for a session.

## Methods

**Fig. 5:**
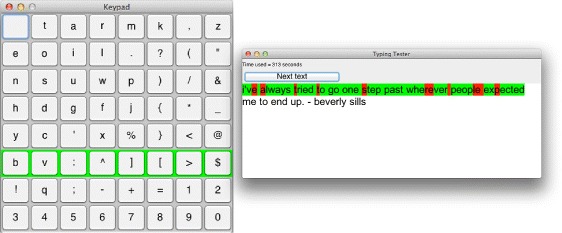
Experiment windows. The prompt and keyboard windows used to gather empirical data on the use of a switch keyboard. The keyboard used a row–column cursor path, and the present location of the cursor is highlighted in *green*. The prompt window indicated what text to enter and provided color feedback as to whether the correct key was entered. The characters are assigned to keys so that the most frequently used characters require few cursor steps and can thereby be quickly reached

We created a typing program with a text window and a virtual keyboard with a row–column cursor path as shown in Fig. 5. The program was run on a computer using Mac OS X with an LCD monitor that has 1920×1200 pixels. Each character entry can be considered to be a trial where the participant’s task is to guide the cursor to the target character on the keyboard by triggering the switch device at the appropriate times. To make the task somewhat engaging, participants were presented with a prompt window that displayed an inspiring quote. The participant’s task was to use the virtual keyboard to type the characters of the quote. To keep the task simple, all characters were in lower case. Throughout the experiment, the assignment of characters to keyboard positions was as given in Fig. 5, which tended to put the most commonly used items at the beginning of the cursor path (Francis & Johnson, 2011).

For each character to be entered, the participant initiated the cursor with a selection action (pressing the switch device), and then the cursor moved across the keyboard with a cursor duration, *D*, that was fixed for a block of sessions. The cursor always started at the top row and moved down the rows of the keyboard. When the participant hit the switch device to select a row, the cursor started to move across the selected row from left to right until the participant again triggered the switch or the cursor went past the last character of the row. A correct selection of the target character caused the prompt window to highlight the target character in green, and an incorrect selection (i.e., the participant responded too early or the cursor moved past the target) led to the target character being highlighted in red in the prompt window (see Fig. 5). There was no opportunity for the participant to correct an error; instead, the trial was scored and the participant started the next trial when ready. When a given quote was finished, the participant clicked the “Next text” button in the prompt window to be presented with another quote. Additional file 5: Movie 5 shows the program in use for a small set of text entry.

The experiment was self-paced, with participants taking breaks as desired and continuing or stopping as motivated. In general, a given session lasted approximately 1 h. Within a session, the cursor duration was fixed, but across sessions it was *D*=0.200, 0.175, 0.150, 0.125, or 0.100 seconds. For each participant, sessions started with the longest cursor durations and the duration was gradually decreased for later sessions. The participant and experimenter together judged whether the participant should move to a faster cursor duration. In a few cases, a participant went back to a slower cursor duration because either they found the new duration to be too short or the wrong cursor duration was accidentally entered for a session. Because of the self-paced and self-motivated properties of the experiment, different participants had a different number of trials for different cursor durations and different numbers of overall sessions.

## Results and discussion

**Fig. 6:**
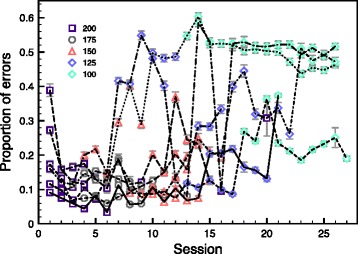
Experimental results. Proportion of entry errors as a function of experimental session. Each curve corresponds to a different participant and different symbols indicate the cursor duration (milliseconds) that was used for that session. Error bars indicate plus and minus one standard error of the proportion. For most statistics, these error bars are quite small because a session involved entering around 1,700 characters

Figure 6 plots the proportion of entry errors as a function of session for each participant, split by the different cursor durations. There is a clear practice effect in the early sessions with the 200-ms cursor duration. It is more difficult to judge whether there are practice effects for other sessions because the cursor duration changes across sessions. As expected, the proportion of errors generally increases as the cursor duration decreases. The exact relationship between cursor duration and proportion of errors varies quite a bit across participants because of the self-paced nature of data collection and the individualized progression to shorter cursor durations. Pooling the character entries across all participants and counting the number of correct and incorrect entries produces the values in Table 1.

**Table 1:** General statistics describing text entry accuracy for the different cursor durations of the experiment

Cursor	Number of	Number of	Proportion of	Proportion of
duration (ms)	characters	entry errors	entry errors	correct entries
200	37,602	4,480	0.119	0.881
175	44,314	4,720	0.107	0.893
150	47,891	7,807	0.163	0.837
125	45,815	12,887	0.281	0.719
100	98,808	41,365	0.419	0.581

**Fig. 7:**
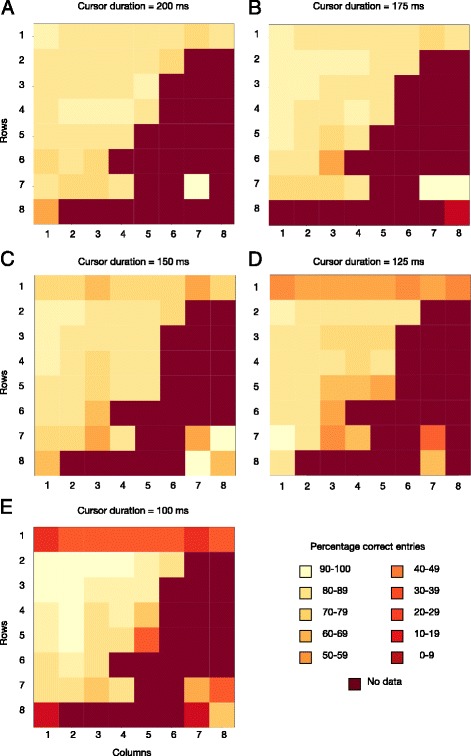
Experimental results. The percentage of correct keyboard entries, combined across all participants, for each row–column keyboard location with the indicated cursor duration

To optimize the design of a switch keyboard, we want to know how entry accuracy varies across the keyboard locations as a function of the cursor duration. Figure 7 color codes the percentage of *correct* entries for each location on the row–column keyboard used in the experiment for each of the tested cursor durations. When the cursor movement is slow (cursor duration is 150–200 ms), Fig. 7 a–c shows that performance is generally above 70 %, although it deteriorates a bit as the cursor duration decreases. Figure 7 d shows that performance notably drops for the 125-ms cursor duration, especially for the first (top) row of the keyboard. Presumably, this characteristic is because the cursor moves past the first row too quickly for the user to hit the switch. Figure 7 e shows that the same behavior is present for the 100-ms cursor duration with even worse performance overall.

### Modeling of error probabilities

To promote optimal switch keyboard designs, we wanted to use the empirical data to develop a model that could predict performance for different cursor paths and different cursor durations. With that goal in mind, we focused on modeling a user’s ability to trigger the switch device at the appropriate times. Each successful trial in the row–column keyboard involves two such triggers: one for selecting the row containing the target and one for selecting the column/key within the selected row. An unsuccessful (error) trial may have one correct or incorrect trigger selection (for selecting a row) but may not have any selection for a column (because there is no need if an error were already made by selecting the wrong row). On some error trials, there may be no selection at all (e.g., the cursor moves past the row containing the target). We hypothesized that trigger timing performance would be a function of the cursor duration and the number of cursor steps from the cursor start or last trigger (e.g., the row or column number).

We deliberately focused the model on the probability of correctly triggering the switch because it enables a predicted calculation of correct entry probabilities for a variety of cursor paths. An important part of this prediction is an independence assumption, meaning that for the same index value of a row or column, the correct selection probabilities are the same. Let *π*_*s*_ be the probability of correctly selecting the switch following *s* cursor steps after the previous selection. For a row–column keyboard, there are two selections, with the first corresponding to the row, *s*=row(*k*), and the second corresponding to the column, *s*=column(*k*), so the error probability of guiding the cursor to position *k* is modeled as the complement of the product of the correct selection probabilities:

5 $$ P_{k} = 1-\pi_{\text{row}(k)} \pi_{\text{column}(k)}.  $$

The same type of probabilities can be used to predict performance for a quadrant cursor path, where the predicted correct entry probability to reach position *k* would be *π*_quadrant(*k*)_ *π*_row(*k*)_ *π*_column(*k*)_ and the predicted error entry probability is 1 minus that product. Similarly, for a linear cursor path, there would be a single opportunity to make an error with probability 1−*π*_*k*_ and for the binary cursor path there would be six opportunities to make errors with associated probabilities.

To estimate the *π*_*s*_ probabilities, we assume they are a function of the cursor duration and the number of cursor steps as defined by logistic regression, that is, the correct selection probabilities can be modeled as follows:

6 $$ \pi_{s} = \frac{\exp(\beta_{0}+\beta_{1}D + \beta_{2}s)}{1+\exp(\beta_{0}+\beta_{1}D+ \beta_{2}s)},  $$

where *s* indicates the number of cursor steps since the last selection action (e.g., the quadrant, row, or column to be selected). The *β* weights were estimated from the experimental data using logistic regression to produce *β*=[−1.85,21.20,0.41]. We also considered a model with an interaction of the cursor duration and the number of cursor steps, but it made the nonsensical prediction that performance deteriorates when the cursor duration increased beyond 400 ms.

**Fig. 8:**
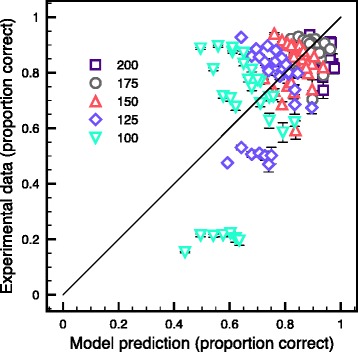
Model fit. Model-predicted and empirically measured proportion of correct keyboard entries. These are combined across all participants for row–column keyboard locations with the indicated cursor duration. Error bars (often too small to be seen) indicate plus/minus one standard error of proportion

To explore the behavior of the model, these parameter estimates were then entered into Eq. (6) to produce estimated correct selection probabilities of $\hat {\pi }_{s}$ for *s*=1,…,8 and cursor durations *D*=0.1, 0.125, 0.150, 0.175, and 0.2 seconds. For the row–column keyboard, the predicted probability of a correct entry is the product of the selection probabilities for the row and column of a target key. To get a sense of how well the model matches the experimental data, Fig. 8 plots the proportion of correct entries for the experimental data against the model-predicted proportion of correct entries for the five experimental cursor durations. Here, only those conditions (key location and duration) with 100 or more trials are included because the model cannot be expected to match the proportions for poorly measured cases. The error bars indicate one standard error for the experimental data (for many points the error bars are smaller than the symbols). Ideally, the points would fall on the diagonal line from bottom left to top right, and while the model is not perfect, the predicted performance does correlate somewhat with the experimental data, *r*=0.57.

**Fig. 9:**
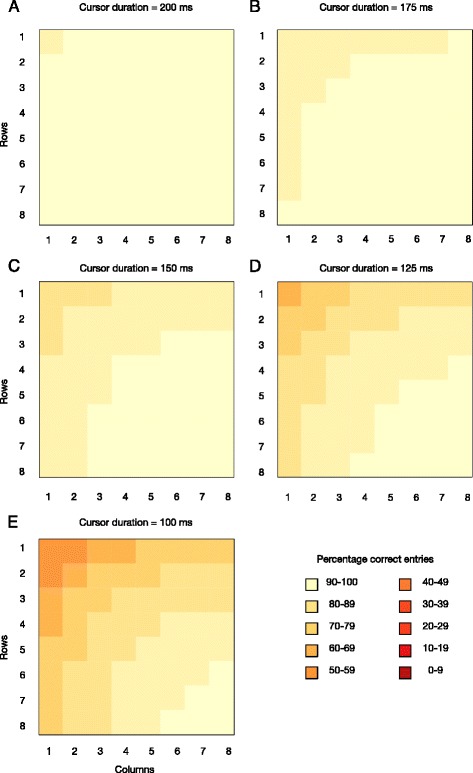
Model performance. Model-predicted percentage of correct keyboard entries for each row–column keyboard location with the indicated cursor duration. The pattern of correct entries is similar to the experimental data in Fig. 7

To understand the model’s behavior further, Fig. 9 displays the predicted correct entry percentages in the same format as in Fig. 7. Unlike the data, the model has no missing conditions, but it generally behaves similarly as the data. In particular, performance deteriorates as the cursor duration decreases. For a cursor duration of 200 ms, there is little variation in performance across the different keyboard locations. For shorter cursor durations, the model predicts improved performance for locations reached with late selections.

Although far from perfect, our overall impression is that the model does a good enough job of accounting for the experimental data that it is fruitful to explore how it contributes to the optimization of switch keyboards. In particular, the model predicts performance for cursor durations and cursor paths that were not measured in the experiment. Hopefully, future research and theories will lead to better model predictions and thus better optimized switch keyboard designs.

### Creating optimized keyboards

To demonstrate the construction of an optimized keyboard, we first design a keyboard for entry of the quotes used in the experiment. Table 2 lists the frequencies, *F*_*i*_, of the characters in the quotes. For a given cursor path, the optimal keyboard is the one that assigns characters to keyboard locations and identifies a cursor duration, *D*, that satisfies *C*_*e*_≤*ε* and minimizes *C*_*t*_.

**Table 2:** Character frequencies for the quotes used in the experiment and for the Python computer code

	Frequency			Frequency			Frequency			Frequency	
Char	Quotes	Python	Char	Quotes	Python	Char	Quotes	Python	Char	Quotes	Python
space	5,346	1,980	p	415	142	:	9	57	} or #	0	30
a	1,764	211	q	19	7	∧	0	0	[	0	65
b	370	54	r	1,385	428	&	0	1	]	0	65
c	800	132	s	1,655	198	*	0	649	-	325	13
d	758	171	t	2,076	271	(	0	113	_	0	91
e	2,876	297	u	827	117	)	0	113	∼	0	0
f	521	76	v	306	48	;	7	0	0	5	100
g	503	48	w	552	42	’	108	70	1	1	80
h	1,138	49	x	23	62	?	15	0	2	2	29
i	1,803	204	y	620	58	+	0	22	3	0	7
j	61	17	z	29	2	=	0	81	4	0	13
k	223	56	!	4	3	&lt;	0	8	5	0	24
l	1,089	109	@	0	1	&gt;	0	3	6	0	19
m	590	167	,	165	101	/	0	21	7	0	12
n	1,680	213	.	434	152	∖	0	2	8	1	4
o	1,995	245	%	2	15	{ or ^″^	0	244	9	1	6

Note that the correct entry probabilities in Eq. (6) depend on the value of the cursor duration, thus the average error rate also depends on the cursor duration. The cursor duration is restricted by the display capabilities of computer hardware. We supposed that a computer monitor refreshes the display at 100 Hz, which means that the shortest cursor duration would be 10 ms and that longer cursor durations would be multiples of 10 ms (covering multiple frames of the display). For a fixed acceptable error rate, we considered all possible cursor durations between 10 and 1,000 ms, in steps of 10 ms. For each of these cursor durations, the MIP algorithm identified the optimal placement of characters to keys to satisfy the acceptable error rate and minimize average search time. The optimal keyboard is the one with the cursor duration that produces the shortest entry time and satisfies the acceptable error rate.

### Optimal row–column keyboards

**Fig. 10:**
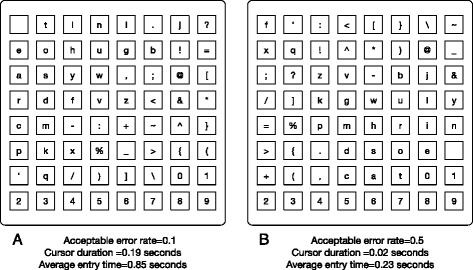
Optimal row-column keyboards. Optimal row–column keyboard design and cursor duration. Acceptable error rates of **a** *ε*=0.1 and **b** *ε*=0.5. This combination of key assignments and cursor duration satisfies the acceptable error rate and minimizes average entry time

We start by considering a row–column keyboard. Figure 10 shows the character arrangements for optimal keyboards that satisfy different acceptable error rates. According to the entry error model, both of these keyboards perfectly match their respective acceptable error rate. Not surprisingly, the optimal cursor duration depends on the acceptable error rate. To satisfy the *ε*=0.1 acceptable error rate, the cursor duration must be rather long, *D*=0.19 seconds (190 ms), while a much shorter cursor duration can be used when the acceptable error rate is *ε*=0.5, *D*=0.02 seconds (20 ms).

To make the average entry time small, one might expect that the optimal design places the most frequent characters early in the cursor path and sets the cursor duration long enough to ensure that the error probabilities for these characters do not become too high. Indeed, this strategy is used for the *ε*=0.1 condition. Here, the most common characters (space, “t”, “e”, “a”, “o”, and “i”) are located along the first few rows and columns of the keyboard. Less frequent characters, such as “&”, “%”, and “]”, are placed at locations that require more cursor steps. This strategy is a good one because the most frequent characters are reached with few steps, which tends to minimize average entry time.

However, an alternative approach is used for the *ε*=0.5 condition. Here the cursor duration is set to be very brief, which means that there is a high probability of making an error for the first few rows and columns. High-frequency characters (“s”, “o”, “e”, “a”, and “t”) tend not to be placed on those error-prone locations and instead are shifted to later rows and columns that have a lower probability of error. The large number of cursor steps needed to reach these frequent keys is offset by the short cursor duration, so the average entry time is low.

Generally, for a fixed acceptable error rate, a decrease in the cursor duration leads to a decrease in average entry time. However, this relationship is not always the case; sometimes increasing the cursor duration led to a decrease in the average search time because an increase in the cursor duration alters the probabilities of making an error and thereby enables a reconfiguration of the characters that leads to faster entry. This kind of effect highlights the need to consider a range of cursor durations and to identify the optimal character assignments to best satisfy the accuracy and speed goals of an optimized keyboard design.

### Optimal keyboard designs for other cursor paths

**Fig. 11:**
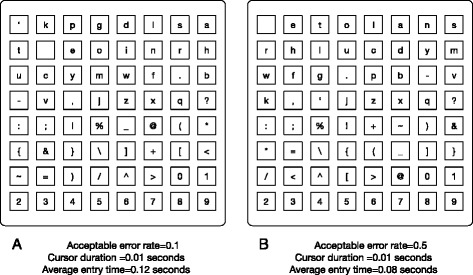
Optimal linear keyboards. Optimal linear keyboard design and cursor duration. Acceptable error rates of **a** *ε*=0.1 and **b** *ε*=0.5. This combination of key assignments and cursor duration satisfies the acceptable error rate and minimizes average entry time

**Fig. 12:**
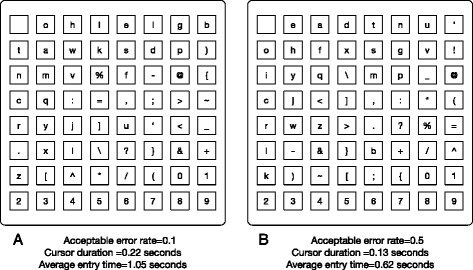
Optimal quadrant keyboards. Optimal quadrant keyboard design and cursor duration. Acceptable error rates of **a** *ε*=0.1 and **b** *ε*=0.5. This combination of key assignments and cursor duration satisfies the acceptable error rate and minimizes average entry time

**Fig. 13:**
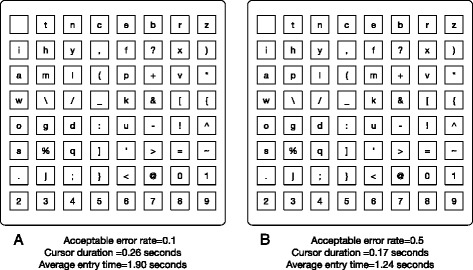
Optimal binary keyboards. Optimal binary keyboard design and cursor duration. Acceptable error rates of **a** *ε*=0.1 and **b** *ε*=0.5. This combination of key assignments and cursor duration satisfies the acceptable error rate and minimizes average entry time

Figures 11, 12 and 13 show the properties of the optimal designs for keyboards using a linear, quadrant, or binary cursor path, respectively. As shown in Fig. 11, a linear cursor path, where the cursor scans over individual keys, generally uses the shortest possible cursor duration (0.01 seconds) and assigns characters to different keys to alter the average error rate and average entry time. For the *ε*=0.5 condition, the optimal keyboard produces an average error rate of 0.35, which is lower than the acceptable error rate of 0.5. This mismatch is because the cursor duration is already at its smallest possible value, and the most frequently used characters are already assigned to their most error-prone locations (e.g., the top row). It simply is not possible for this cursor path to further trade off accuracy for speed for this text corpus.

Figure 12 shows two optimal quadrant keyboards, where the cursor first moves over different quadrants and then rows and columns within a selected quadrant. Both keyboards tend to place high-frequency characters in the first two quadrants (top-left and top-right quadrants). They differ in the placement of low-frequency characters and in the cursor duration, with the keyboard requiring a low acceptable error rate having a cursor duration nearly twice as long as the keyboard requiring a high acceptable error rate.

Figure 13 shows two optimal binary keyboards, where the cursor alternates between remaining halves of the keyboard to zero in on the desired key. Here, a change in the acceptable error rate from 0.1 to 0.5 hardly alters the assignment of characters to keys (only the “m” and “p” characters switch positions), which makes some sense because different key positions are nearly equivalent due to the way the cursor focuses in on a target key. For example, with a binary cursor path, 20 of the 64 keys are reached with nine cursor steps, while an additional 30 keys are reached with eight or ten cursor steps. With such homogeneity, changing the key assignments for a binary cursor path often has very little impact on the average entry time. In a similar way, key assignments have only a modest effect on the error entry rate. Thus, the main variable controlling speed and accuracy for a binary cursor path is the cursor duration, which is longer for smaller acceptable error rates.

**Fig. 14:**
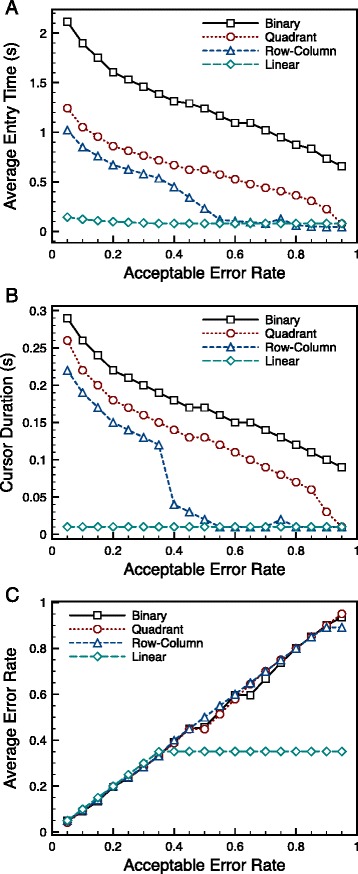
Properties of optimal keyboards. Properties of optimal keyboards for different cursor paths as a function of the acceptable error rate. **a** A comparison of average entry time indicates that the linear keyboard is predicted to perform best for most acceptable error rates. **b** The cursor duration of the optimal keyboard for each cursor path indicates that the linear keyboard uses a nearly constant cursor duration at the minimum possible value of 0.01 seconds. **c** All keyboards except for the linear keyboard trade off accuracy for speed by producing a predicted average error rate that is close to the acceptable error rate

A user of a switch keyboard wants to identify the best possible keyboard design for the type of text entry they will use, and for an acceptable error rate. As described above, the optimal design depends on the cursor path, so users should compare the optimal keyboard designs for different cursor paths and choose the best. Figure 14 summarizes the properties of optimal keyboard designs for different cursor paths as a function of the acceptable error rate. Critically, Fig. 14 a indicates that for all but the highest acceptable error rates, the linear cursor path produces a much smaller average entry time than any other cursor path. This advantage for the linear cursor path is because it involves a single switch action, so there is only one opportunity for the user to make an entry error. In contrast, the row–column cursor path always involves two switch actions, and the quadrant and binary cursor paths have more switch actions and therefore more opportunities for an entry error. The linear cursor path leverages this advantage by making the cursor duration extremely brief and assigning characters to keys in a way that satisfies the acceptable error rate.

It is notable that the optimal solution for a linear cursor path does not always look like a very good keyboard design. For example, the keyboard in Fig. 11 b places the space character at the first key. The logistic model predicts that a user has a 0.77 probability of making an entry error at this key, so this most commonly used character is unlikely to be entered correctly. Such a property seems to make the keyboard essentially unusable, but this limitation simply reflects the requirements imposed on the design. A keyboard with an acceptable error rate of 0.5 is, indeed, unlikely to be a usable keyboard. The problem is not with the design process but with the design specification. There is a similar problem for the keyboard in Fig. 11 a, which satisfies an acceptable error rate of 0.1. Here, the “k” character is on the second key, which has a predicted error rate of 0.7. Clearly, someone entering the word *knock* on such a keyboard faces low odds of success. But for the provided text corpus (the quotes used in the experiment), the letter “k” is rather uncommon (Table 2), so this type of entry is uncommon. One could impose additional constraints on the design process, such as to ensure that every key has an acceptable error rate in addition to an acceptable average error rate. The optimization process would be similar to what is described above, but the additional constraint would result in quite different optimized keyboards.

### Effect of a different text corpus on optimal keyboard design

The optimal keyboard design also depends on the distribution of character frequencies *F*_*i*_, which are defined by the text corpus that is appropriate for a given user. These frequency distributions may vary widely depending on what kind of material is being entered into the keyboard. For example, a user who writes novels or poems may rarely use characters such as “[” or “}”, while a user who writes Python computer programs would commonly use characters such as “i” and “f” as well as “(” and “*”. To explore the effect of the text corpus on optimal keyboard design, we identified the frequency of 64 characters from an early version of Python code that was used to calculate the optimized keyboard, and we compared the resulting keyboards to the keyboards designed for the quotations used in the experiment. Table 2 shows the frequencies of the characters included in our Python code. To ensure that all Python-related characters were part of the keyboard, the characters “{” and “}” in the original keyboard were replaced by the characters “#” and “ ^″^”.

**Fig. 15:**
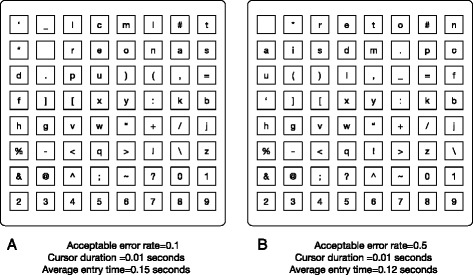
Python-based optimal linear keyboards. Optimal linear keyboard design and cursor duration relative to the Python code text corpus. Acceptable error rates of **a** *ε*=0.1 and **b** *ε*=0.5. This combination of key assignments and cursor duration satisfies the acceptable error rate and minimizes average entry time

Figure 15 shows optimal keyboards using a linear cursor path for the Python text corpus. Like the linear cursor path keyboards for the quotes, the cursor duration is at the smallest possible value, 0.01 seconds. Given the differences in character frequencies, it is not surprising that the optimized assignment of characters to keyboard positions varies with the text corpus, as a comparison of Figs. 11 and 15 demonstrates. Nevertheless, the same basic principles underlie each optimized keyboard. For a low acceptable error rate, the high-frequency characters are placed a bit down the cursor path so that they have a low probability of an entry error and low-frequency characters are placed at the beginning of the cursor path. For a higher acceptable error rate, the high-frequency characters are placed at the beginning of the cursor path, where the keys have the highest predicted entry error probability and are reached most quickly.

The average entry time seems to be faster for the keyboards optimized for the quotes text than the keyboards optimized for the Python code text. This difference reflects the distribution of frequencies across the characters in the text corpus. Among other things, the Python computer code has 294 uses of numerals, while the quotes text uses numerals only ten times. Since the numerals are all fixed at the end of the linear cursor path, they take a relatively long time to reach.

For completeness, we also investigated the relationships between average entry time, cursor duration, average error rate, and the acceptable error rate for different cursor paths, using the Python code text corpus. These relationships are very similar to the results obtained for the quotes text corpus shown in Fig. 14.

## Conclusions

People with severe motor control problems often rely on switch devices to communicate. We studied the fundamental characteristics of a switch virtual keyboard by framing the design task as a speed/accuracy trade-off optimization problem. In particular, we proposed a MIP model for this problem to minimize the average entry time, given an acceptable error threshold. We further described how to estimate and model the necessary error probabilities. Automating these optimization tasks can be part of a general method for improving the usability of these kinds of devices (Koester & Simpson, 2014).

The approach considers many needs of the user. First, the acceptable error rate is established by the user, which allows them to specify their own typing style. Second, the user can choose their own text corpus depending on their personal interests and needs. We also tested the effects of different cursor paths, and showed that the optimization process selects an optimal keyboard for different cursor paths by balancing the trade-off between the average entry time and the average error rate.

A consistent outcome is that an optimized keyboard with a linear cursor path is much faster than an optimized keyboard for other cursor paths. This result is surprising because virtual keyboards on the market generally use a hierarchical structure similar to a quadrant cursor path, and often have even more levels to the hierarchy. The optimization analysis presented here suggests that a deep hierarchy is problematic because it introduces many opportunities for an error. To compensate for the high risk of error, the cursor duration has to be made so long that the average entry time is quite large. A linear cursor path avoids these problems by requiring only one selection. When coupled with a short cursor duration, the average entry time for a linear cursor path can be quite short.

However, there are two important limitations to the current analysis that suggest additional investigation is required before broadly recommending linear cursor paths. First, the efficiency of a linear cursor path depends on the frequency distribution of characters in the text corpus. When more characters are part of the keyboard, the number of cursor steps for characters at the end of the path increases accordingly. If the frequency distribution of characters is fairly uniform, then average entry time will dramatically increase for larger keyboards, and there may then be an advantage to hierarchical cursor paths. Second, the predicted optimal linear keyboards used the smallest possible cursor duration (10 ms), but the model prediction of performance for such a cursor duration is based on experimental data that measured performance for much longer cursor durations (the briefest being 100 ms). We do not have a lot of confidence in the model’s prediction for such short cursor durations. In a similar way, the predicted performance for cursor steps beyond eight are also not directly supported by experimental data. We do not have evidence that the model does poorly in these conditions, but there should be validation of the model (and possibly refinement) with additional empirical studies.

The optimization approach can be elaborated to consider additional characteristics of keyboard function. For example, a popular approach is to enable predictive typing, where the keyboard monitors what is typed and presents a list of options and the user can select a full word after entering the first few letters. Such a feature carries both positive and negative aspects for users of switch keyboards. On the positive side, it may require entering fewer characters to type common words and thereby it may speed communication. On the negative side, navigating to the list of choices requires the user to guide the cursor to the list (either with a linear cursor path or in a hierarchical structure). There is, of course, a risk of a selection error when trying to reach an item in the prediction list, and an error in that context may alter the list and thereby render the system rather difficult to work with. If a prediction system is part of a keyboard, then there is a need to optimize its design within the keyboard, and the approach we have presented here can be modified to provide such optimization. Similarly, it is important to consider how users would correct errors with a switch keyboard. Error correction with a switch keyboard using a delete or undo command can only be initiated by the user guiding the cursor to an appropriate action key. To correct past text, switch keyboards have action keys that move a marker in the text (e.g., up a line or down a page). Some switch keyboards also allow for control of mouse actions (movement, hold, drag, select, stop, click, and double-click commands), which can be used to fix errors, among other things. Such command keys should also be optimized according to their frequency of use for a given user.

Future work should also consider details of the switch device. Different patients are able to use different muscles to control a switch device. A person who uses eye blinks (Bauby, 1997), a sip-puff headset, or manipulates EEG signals (Serby, Yom-Tov & Inbar, 2005) may produce very different error probabilities than what is reported here and thus would require different keyboard designs. Future work should also explore easier methods of estimating and modeling a user’s ability to use the switch device. Koester & Simpson (2014) simply measured how well users could trigger single, double, and triple clicks of the switch device. Such an approach would be much faster than the empirical approach used here. Likewise, Simpson, Koester and LoPresti (2007) proposed that an appropriate cursor duration is one that generates a ratio of the user’s reaction time and the cursor duration equal to 0.65. If true in general, then it should be easy to model error probabilities. Regardless of the empirical method, once the error probabilities for a user’s ability with a switch device are identified and modeled, the optimization algorithm described here can be readily applied.

Although our investigation was motivated by the needs of locked-in patients using switch keyboards, the computational optimization approach developed here should also apply to other users and keyboards. For example, touchscreen devices in automobiles (including keyboards) face a similar entry constraint (e.g., drivers and passengers tend to use single-finger entry rather than multiple-finger typing) and time-sensitive environments (e.g., Young Lee, Gibson & Lee, 2016). Although the interface method is rather different, there remains a speed/accuracy trade-off that can be optimized using some of the methods reported here. Military helicopter pilots face similar design issues when interacting with a multifunction display (Francis, 2000; Francis & Rash, 2005). Thus, basic research into the design of switch keyboards for locked-in patients should prove fruitful in addressing the design of other types of systems that face fundamentally similar problems despite substantial differences in users, use cases, and interface details.

## Additional files

Additional file 1 Movie 1. Animation of a switch keyboard where the cursor follows a linear path across the keyboard. The “Hit switch” text below the virtual keyboard indicates when a user would need to trigger a switch device to guide the cursor toward a target letter (“k” in this case). The cursor duration is *D*=200 ms. (GIF 387 kb)

Additional file 2 Movie 2. Animation of a switch keyboard where the cursor follows a row–column path across the keyboard. The “Hit switch” text below the virtual keyboard indicates when a user would need to trigger a switch device to guide the cursor toward a target letter (“k” in this case). The cursor duration is *D*=500 ms. (GIF 211 kb)

Additional file 3 Movie 3. Animation of a switch keyboard where the cursor follows a quadrant path across the keyboard. The “Hit switch” text below the virtual keyboard indicates when a user would need to trigger a switch device to guide the cursor toward a target letter (“m” in this case). The cursor duration is *D*=750 ms. (GIF 214 kb)

Additional file 4 Movie 4. Animation of a switch keyboard where the cursor follows a binary path across the keyboard. The “Hit switch” text below the virtual keyboard indicates when a user would need to trigger a switch device to guide the cursor toward a target letter (“*” in this case). The cursor duration is *D*=750 ms. (GIF 310 kb)

Additional file 5 Movie 5. A movie of the switch keyboard typing program used in the experiment. The user clicks the mouse (a switch device) to guide the cursor (green highlighting on the virtual keyboard) toward the next letter in the quote. Color-coded feedback indicates whether the user was successful (green) or not (red) in selecting the desired character. Note that the cursor duration here (400 ms) is much longer than what was used in the experiment. (MP4 7137 kb)
