# Supplementary figures and images for: How to optimize switch virtual keyboards to trade off speed and accuracy
Source: Cogn Res Princ Implic. 2016 Sep 22;1:6. doi: 10.1186/s41235-016-0007-6 (PMC5256434; doi:10.1186/s41235-016-0007-6)

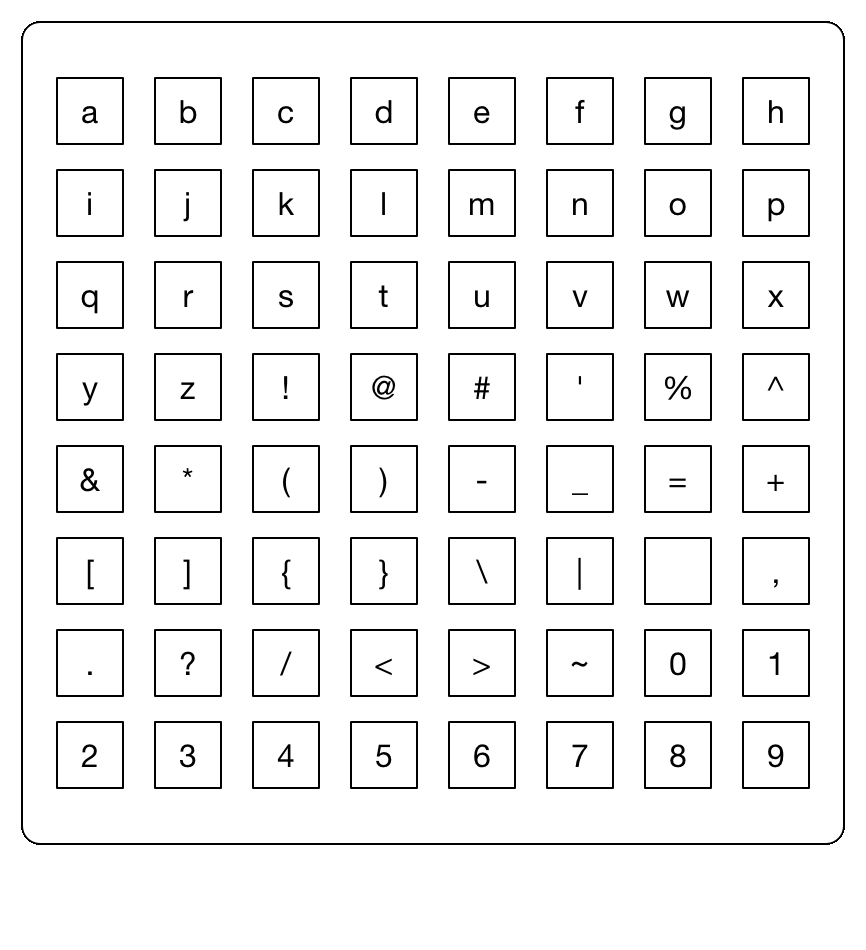

Supplement: Supplementary file 1 — Movie 1. Animation of a switch keyboard where the cursor follows a linear path across the keyboard. The “Hit switch” text below the virtual keyboard indicates when a user would need to trigger a switch device to guide the cursor toward a target letter (“k” in this case). The cursor duration is D=200 ms. (GIF 387 kb) [file 41235_2016_7_MOESM1_ESM.gif]

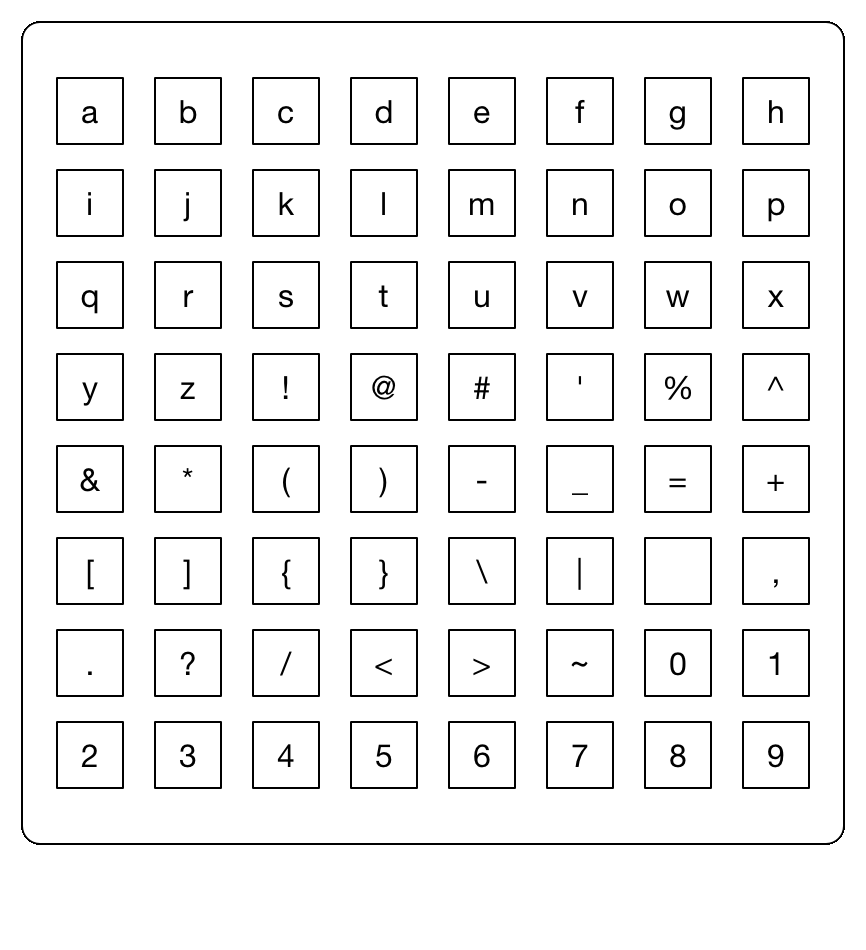

Supplement: Supplementary file 2 — Movie 2. Animation of a switch keyboard where the cursor follows a row–column path across the keyboard. The “Hit switch” text below the virtual keyboard indicates when a user would need to trigger a switch device to guide the cursor toward a target letter (“k” in this case). The cursor duration is D=500 ms. (GIF 211 kb) [file 41235_2016_7_MOESM2_ESM.gif]

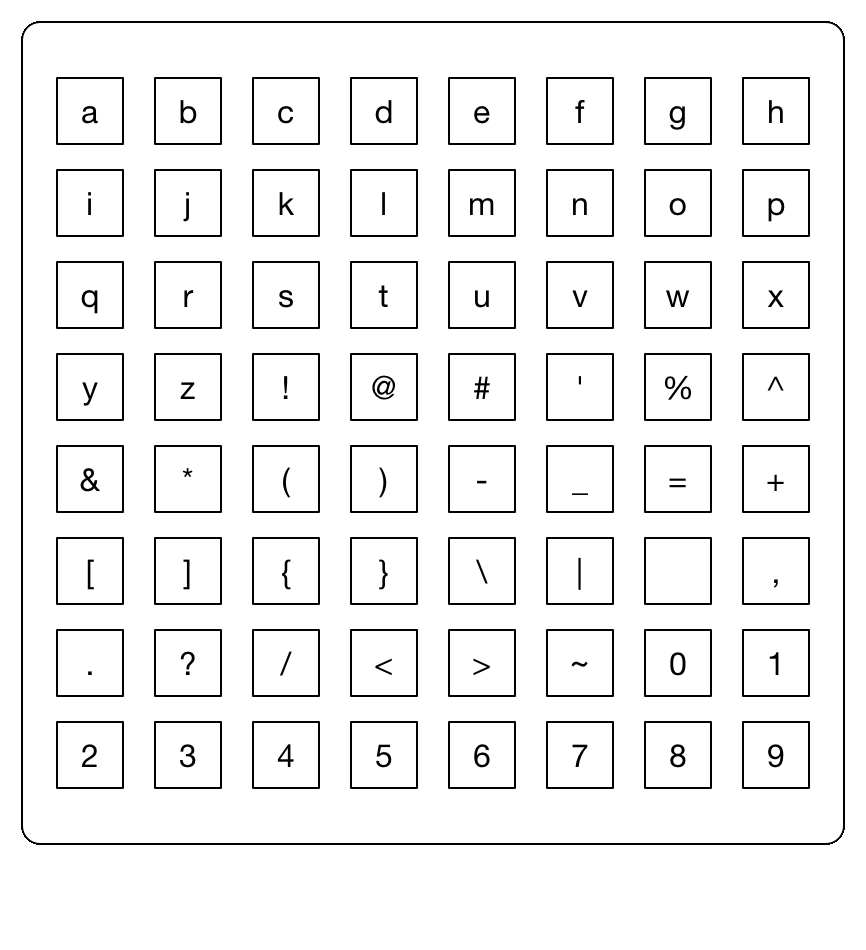

Supplement: Supplementary file 3 — Movie 3. Animation of a switch keyboard where the cursor follows a quadrant path across the keyboard. The “Hit switch” text below the virtual keyboard indicates when a user would need to trigger a switch device to guide the cursor toward a target letter (“m” in this case). The cursor duration is D=750 ms. (GIF 214 kb) [file 41235_2016_7_MOESM3_ESM.gif]

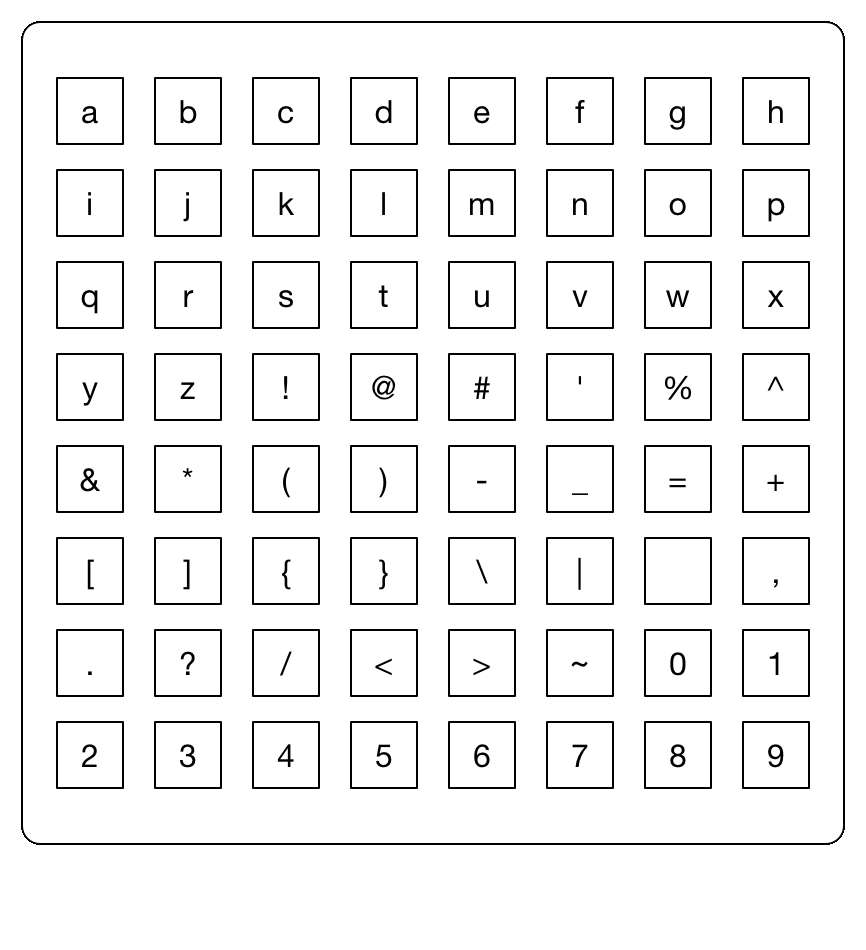

Supplement: Supplementary file 4 — Movie 4. Animation of a switch keyboard where the cursor follows a binary path across the keyboard. The “Hit switch” text below the virtual keyboard indicates when a user would need to trigger a switch device to guide the cursor toward a target letter (“*” in this case). The cursor duration is D=750 ms. (GIF 310 kb) [file 41235_2016_7_MOESM4_ESM.gif]
